# Binding Kinetics of Ruthenium Pyrithione Chemotherapeutic Candidates to Human Serum Proteins Studied by HPLC-ICP-MS

**DOI:** 10.3390/molecules25071512

**Published:** 2020-03-26

**Authors:** Katarina Marković, Radmila Milačič, Stefan Marković, Jerneja Kladnik, Iztok Turel, Janez Ščančar

**Affiliations:** 1 Department of Environmental Sciences, Jožef Stefan Institute, Jamova 39, SI-1000 Ljubljana, Slovenia; katarina.markovic@ijs.si (K.M.); radmila.milacic@ijs.si (R.M.); stefan.markovic@ijs.si (S.M.); 2 Jožef Stefan International Postgraduate School, Jamova 39, SI-1000 Ljubljana, Slovenia; 3 Faculty of Chemistry and Chemical Technology, University of Ljubljana, Večna pot 113, SI-1000 Ljubljana, Slovenia; Jerneja.Kladnik@fkkt.uni-lj.si

**Keywords:** ruthenium-based chemotherapeutics, drug candidates, pyrithione, kinetics study, human serum, speciation analysis, CLC monolithic chromatography, CIM Protein G and DEAE disks, UV spectrometry, isotope dilution inductively coupled plasma mass spectrometry

## Abstract

The development of ruthenium-based complexes for cancer treatment requires a variety of pharmacological studies, one of them being a drug’s binding kinetics to serum proteins. In this work, speciation analysis was used to study kinetics of ruthenium-based drug candidates with human serum proteins. Two ruthenium (Ru) complexes, namely [(η^6^-*p*-cymene)Ru(1-hydroxypyridine-2(1*H*)-thionato)Cl] (**1**) and [(η^6^-*p*-cymene)Ru(1-hydroxypyridine-2(1*H*)-thionato)pta]PF_6_ (**2**) (where pta = 1,3,5-triaza-7-phosphaadamantane), were selected. Before a kinetics study, their stability in relevant media was confirmed by nuclear magnetic resonance (NMR). Conjoint liquid chromatography (CLC) monolithic column, assembling convective interaction media (CIM) protein G and diethylamino (DEAE) disks, was used for separation of unbound Ru species from those bound to human serum transferrin (Tf), albumin (HSA) and immunoglobulins G (IgG). Eluted proteins were monitored by UV spectrometry (278 nm), while Ru species were quantified by post-column isotope dilution inductively coupled plasma mass spectrometry (ID-ICP-MS). Binding kinetics of chlorido (**1**) and pta complex (**2**) to serum proteins was followed from 5 min up to 48 h after incubation with human serum. Both Ru complexes interacted mainly with HSA. Complex (**1**) exhibited faster and more extensive interaction with HSA than complex (**2**). The equilibrium concentration for complex (**1**) was obtained 6 h after incubation, when about 70% of compound was bound to HSA, 5% was associated with IgG, whereas 25% remained unbound. In contrast, the rate of interaction of complex (**2**) with HSA was much slower and less extensive and the equilibrium concentration was obtained 24 h after incubation, when about 50% of complex (**2**) was bound to HSA and 50% remained unbound.

## 1. Introduction

Cancer is a noncommunicable disease, which is, due to an increase in life expectancy, the leading cause of morbidity worldwide. In 2018, there were approximately 18.1 million new cancer cases worldwide. Cancer was the cause of death for 9.6 million people [1]. Chemotherapy is the most common cancer treatment in which platinum-based chemotherapeutics are widely used. Among them, cisplatin, carboplatin and oxaliplatin have been approved by relevant regulatory institutions like the United States Food and Drug Administration (FDA), for the treatment of various cancers [2,3,4,5]. However, the commonly used platinum-based chemotherapeutics are also associated with induced drug resistance [6] and severe side effects like nephrotoxicity, ototoxicity, emetogenesis and neurotoxicity [7]. To minimize side effects and to reduce drug resistance, new platinum-based chemotherapeutics were synthesized [2,8] and combined modality regimes of cancer therapy like electrochemotherapy were introduced [9,10]. In advanced targeted therapies of cancer, various drug carriers, like nanocarriers, have also been applied [11]. Increased activities are carried out towards the development and improvement of other metal-based compounds and non-classical platinum (Pt) complexes with enhanced safety and cytotoxic profile [12,13]. As one of the most promising alternatives to platinum-based chemotherapeutics are Ru(II) and Ru(III) complexes. They possess unique multifunctional biochemical properties and exhibit lower toxic effects than platinum-based chemotherapeutics [14,15]. In cancer cells, trivalent Ru complexes are speculated to be reduced by cellular reductants, e.g., ascorbates, to their more active divalent form. For the majority of Ru complexes, the cellular mode of action differs from the DNA-binding mechanism, which is typically associated with platinum-based chemotherapeutics [16]. Despite the wide range of intracellular targets, only two Ru complexes—KP1339, which is the sodium salt analogue of KP1019 (indazolium trans-[tetrachlorobis(1*H*-indazole)-ruthenate(III)]) and TLD1433 ([Ru(II) (4,4′-dimethyl-2,2′-bipyridine)2(2-(2′,2″:5″,2‴-terthiophene)-imidazo[4,5-f])]Cl_2_)—are currently in the clinical trials [2,15,16,17]. While Ru(III) complexes KP1339 have been developed as chemotherapeutic agents, the octahedral Ru(II) complex TLD1433 exhibits potential for photo-dynamic therapy [16]. Moreover, organoruthenium(II)-arene-pta (RAPTA)-type compounds have turned out to be potent cytotoxic and antiangiogenic agents and therefore, they represent an interesting class of Ru compounds to be further investigated [18].

**Figure 1:**
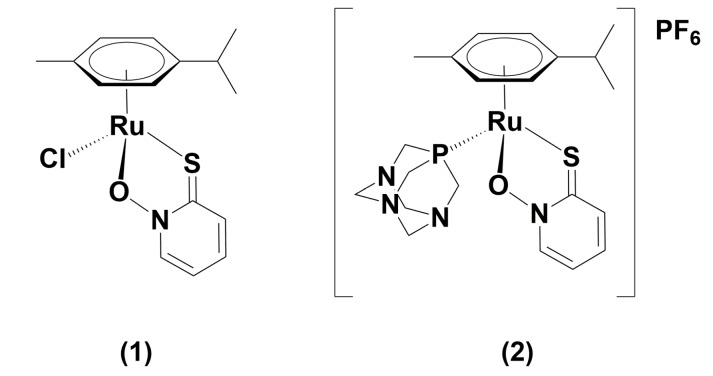
Chemical structures of [(η^6^-*p*-cymene)Ru(1-hydroxypyridine-2(1*H*)-thionato)Cl] (**1**) and [(η^6^-*p*-cymene)Ru(1-hydroxypyridine-2(1*H*)-thionato)pta]PF_6_ (**2**).

Recently, in the Turel group, η^6^-*p*-cymene Ru(II) chlorido coordination compound with ligand pyrithione (2-mercaptopyridine *N*-oxide) (1) (Figure 1) was synthetized and examined for its anticancer activity [19]. The organoruthenium(II) pyrithione complex exhibits promising anticancer potential without cytotoxicity towards non-cancerous cells. It also shows an inhibitory effect towards glutathione-S-transferase (GST), the key enzyme involved in the development of drug resistance in cancer treatment [20]. The research was further oriented to the development of new chlorido and 1,3,5-triaza-7-phosphaadamantane (pta) organoruthenium(II) analogue complexes with methyl-substituted pyrithiones. The stability of these complexes was studied under biologically relevant conditions. They were tested for their cytotoxicities against several cancer cell lines and one normal cell line. Their mode of action was thoroughly assessed through wound healing assay, binding to bovine serum albumin (BSA), induction of apoptosis, cell cycle analysis, DNA interactions, generation of reactive oxygen species (ROS), inhibition of the potential molecular target thioredoxin reductase (TrxR) and mitochondrial function assay [21]. 

An important step in a drug candidate’s characterization is the pharmacological evaluation of its interactions with serum proteins. It provides data for the elucidation of the mechanism of antitumor therapy, the behaviour of the intact drug, its biotransformation in clinical samples at physiologically relevant concentrations and, as the final goal, its dosage adjustment. This information can be largely acquired by a speciation analysis [22,23,24,25,26,27]. For a metalloprotein speciation analysis, the most frequently applied was high-performance liquid chromatography (HPLC) hyphenated to inductively coupled plasma mass spectrometry (ICP-MS) detector. Among HPLC techniques, size exclusion chromatography (SEC) in combination with ICP-MS was used for speciation of metalloproteins [28] and in the investigations of serum binding properties of anticancer agents [29]. However, the low resolution of the SEC, which does not always allow selective separation of proteins, and possible irreversible binding of proteins to the column stationary phase, are the major limitations of this technique. A moderately long time of the analytical run in SEC analysis is a limiting factor for kinetic studies as well [28]. Hence, to overcome stated difficulties, anion-exchange chromatography, available as particle packed columns and monolithic columns, was applied. Monolithic chromatography has several advantages over particle packed chromatography. Among them are the improved protein separation due to efficient mass transport and the use of higher flow rates. This shortens the time of analysis, allowing implementation of kinetic studies [30,31,32]. Monolithic columns are also more robust and enable analyses of much larger series of serum samples than particle packed columns [33]. Another great advantage of monolithic columns is the possibility to modify their stationary phases. This results in different biologically active phases, which are then implemented in affinity monolithic chromatography. Since different types of monolithic supports are available as disks, they can be assembled together into conjoint liquid chromatography (CLC) column, which widens options of their use in speciation analysis [34,35,36,37]. In our group, this analytical approach has been applied in investigations of the kinetics of Pt-based chemotherapeutics [34,35] and Ru-based drug candidates [36] in human serum, and in speciation analyses of serum of cancer patients treated with cisplatin or carboplatin [35]. In these studies, convective interaction media (CIM) protein G and anion-exchange diethylamino (DEAE) monolithic disks were placed in one housing, forming conjoint liquid chromatography (CLC) monolithic column, which enabled two-dimensional separation in a single chromatographic run. For accurate quantification of the separated Pt or Ru species, the post-column isotope dilution (ID)-ICP-MS technique was applied. A similar methodology was used in the group of Goenaga-Infante [37] for speciation of carboplatin adducts with serum proteins. In their research, separated Pt species were quantified by species-specific ID-ICP-MS. 

In the investigations of performances of new drug candidates, it is important to elucidate the kinetics of drug bindings to human serum proteins. Therefore, the aim of the present study was to apply the speciation analysis to investigate the kinetics of two prosperous Ru-based anticancer drug candidates in human serum, namely Ru(II) chlorido [(η^6^-*p*-cymene)Ru(1-hydroxypyridine-2(1*H*)-thionato)Cl (**1**) and Ru(II) pta [(η^6^-*p*-cymene)Ru(1-hydroxypyridine-2(1*H*)-thionato)pta]PF_6_ complex (**2**), synthesized in Turel group [19,21]. Kladnik et al. [21] have already reported some preliminary results on protein binding of analogue methyl-substituted pyrithione Ru(II) chlorido and pta complexes performed on bovine serum albumin (BSA) by atomic absorption spectroscopy. It was shown that both chlorido and pta complexes bind to BSA but in different amount. In order to get deeper insight into the binding kinetics of complexes (**1**) and (**2**), Ru species were separated on CLC column composed of CIM Protein G and anion-exchange DEAE disks. Separated serum proteins were detected by UV spectrometry and separated Ru species were quantified by post-column ID-ICP-MS. Serum samples were spiked with chlorido (**1**) and its pta analogue complex (**2**) and kinetics of binding to serum proteins were followed by the speciation analysis from 5 min up to 48 h after spiking.

## 2. Results and Discussion

### 2.1. Stability of [(η^6^-p-Cymene)Ru(1-Hydroxypyridine-2(1H)-Thionato)Cl (1) and [(η^6^-p-Cymene)Ru(1-Hydroxypyridine-2(1H)-Thionato)pta]PF_6_ (2) and Their Behaviour in Aqueous Solutions

The chemical structures of complexes (**1**) and (**2**) used in the present study are presented in Figure 1.

The stability of complexes (**1**) and (**2**) was tested by the ^1^H and ^31^P-NMR spectroscopies, which enable tracking of structural changes in the investigated media. Isolated chlorido complex (**1**) is a neutral Ru(II) species, where positive charge of the metal ion is compensated by negative charges of the ligand pyrithione and chloride anion. However, it is well known that such species, when in contact with aqueous media, exchange their halide ion with neutral water molecules and become positively charged and therefore activated through hydrolysis. These cationic complexes can more easily interact with biological molecules and trigger pharmacological effect [38]. On the other hand, isolated pta complex (**2**) is an ionic-type compound. It consists of positively charged complex cation in which neutral pta and anionic pyrithione ligands are bound to Ru(II), whereas PF_6_^−^ as a counter anion is needed to ensure electrochemical neutrality. In aqueous solutions, PF_6_^−^ dissociates from complex (**2)**, but in contrast to complex (**1**), no ligand attached to Ru is easily replaced. Spectra were recorded in 0.3% MeOD-d_4_/D_2_O solution containing 154 mM NaCl or D_2_O solution containing 154 mM NaCl for complex (**1**) or (**2**), respectively. Media for each complex were chosen according to their sample preparations for kinetics studies (see Section 3.3). NaCl was used to mimic chloride concentration in human serum to examine the possible influence of high extracellular concentration of that ion. As reported, a high concentration of NaCl suppresses hydrolysis of the chlorido form Ru-Cl, thus, hydrolyzed Ru-OH_2_ or Ru-OH species are probably not present in the extracellular medium or at least in a lower percentage. However, the Cl^-^ concentration inside the cells is lower so the hydrolysis is more likely to occur there and activated complexes can trigger further biological effect [39]. Contrarily to Ru-Cl complexes, Ru-pta compounds with bidentate ligands were reported to resist hydrolysis so that the administration of the drug in saline with high NaCl concentration would not be necessary. However, the choice of the bidentate ligands for Ru-pta complexes must be carefully chosen as ligands bound too strongly to Ru(II) could be inactive, whereas ligands bound too loosely could be easily hydrolyzed to mentioned aqua species [40]. The NMR spectra for complexes (**1**) and (**2**) were recorded immediately after the dilution, and after 24 h and 48 h, which was the last time point for speciation analysis of kinetic studies (Figures S1–S3). Small changes of complex (**1**) can be observed in ^1^H-NMR spectra, when a minor amount of *p*-cymene ring is released after 24 h (Figure S1). The concentration of the latter slightly increases until the second day. Similarly, it was observed for pta complex (**2**), where also small amount of pta oxide (ptao – 1,3,5-triaza-7-phosphaadamantane-7-oxide) can be detected (Figure S2). Still, investigated Ru(II) chlorido as well as pta species remain the main compounds present. Importantly, no visible changes for pta complex (**2**) in ^31^P-NMR spectra were monitored (Figure S3). The obtained data are also in good agreement with previously reported stabilities for such systems [19,21].

### 2.2. Optimization of the Analytical Procedure for Speciation of Ru Complexes *(**1**)* and *(**2**)* on the CLC Column

**Figure 2:**
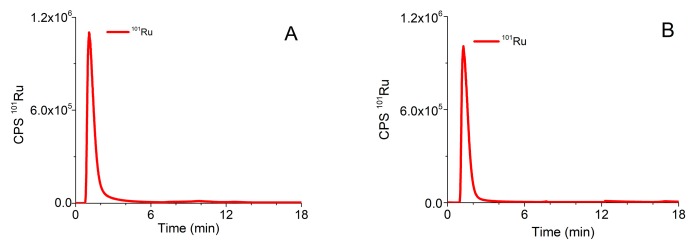

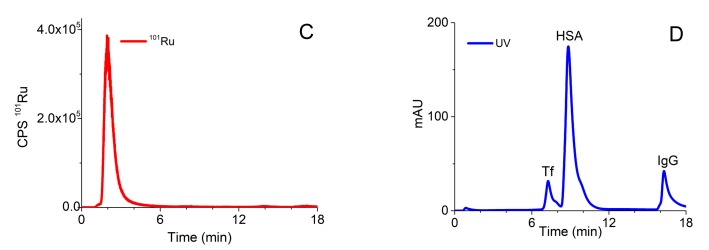
Typical chromatograms of separation of (**A**) Ru3+ (2 µg/mL Ru), (**B**) complex (**1**) (1.64 µg/mL Ru), (**C**) complex (**2**) (1.60 µg/mL Ru) followed by ICP-MS detection at *m*/*z* 101, and (**D**) 5-times diluted mixture of standard serum proteins (25 g/L HSA, 5 g/L IgG and 2.5 g/L Tf) monitored by UV spectrometry at 278 nm.

A procedure that was previously applied for speciation of Pt-based chemotherapeutics on the CLC column, which enabled two-dimensional separation in one chromatographic run [34], was optimized for the investigation of speciation of the Ru-drug candidates. The optimized analytical procedure is described in Section 3.5. Typical chromatograms of separation of ionic Ru (Ru^3+^), Ru complexes (**1**) and (**2**) monitored by ICP-MS at *m/z* 101, and of the mixture of serum proteins followed by UV spectrometry (278 nm) detection on the CLC column are presented in Figure 2.

In order to prevent partial adsorption of complex (**2**) on the stationary phase of the CLC column and to enable separation of unbound Ru species from those bound to serum proteins, 10 mM NH_4_Cl was added to buffer A and isocratic elution with buffer A was applied for 5 min. As can be seen from Figure 2, Ru^3+^ and positively charged complexes (**1**) and (**2**) pass through the Protein G and CIM DEAE disks and are eluted prior to the elution of main serum proteins. During gradient elution, from 100% buffer A to 50% buffer B, Protein G disk retained IgG, while Tf and HSA were separated on the DEAE disk. Finally, IgG was rinsed from the column by isocratic elution with 0.5 M AcOH. Data from Figure 2 revealed that the optimized speciation procedure enables the effective separation of positively charged Ru species from serum proteins.

**Figure 3:**
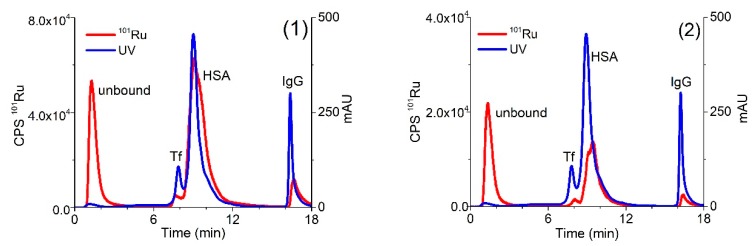
Two-dimensional separation of 5-times diluted human serum spiked with complexes (**1**) (0.760 µg/mL Ru) or (**2**) (0.396 µg/mL Ru) on CLC monolithic column 24 h after incubation, followed by UV spectrometry at 278 nm and ICP-MS at *m/z* 101 detection.

In the following experiments, human serum sample was spiked with solutions of complexes (**1**) or (**2**) and a speciation analysis on the CLC column was performed 24 h after incubation at 37 °C. The separation of proteins was followed by UV spectrometry at 278 nm and monitored by ICP-MS at *m/z* 101 for Ru species (Figure 3).

As evident from the data of Figure 3, the optimized speciation procedure enables the separation of positively charged Ru species (unbound Ru (**1**) and (**2**) complexes) and their adducts with main serum proteins. As mentioned in Section 2.1, presented Ru-Cl and Ru-pta complexes (Figure 1) can undergo hydrolysis; therefore, their Ru-OH and Ru-OH_2_ species in unbound fraction might be present in the solution as well. Apart from mentioned hydrolysis products, there is a possibility of interference of Ru complexes with some low molecular mass components from serum, too. We are aware of the importance of the identification of the mixture of Ru species that might be present in the solution. However, in this study, we focused on the kinetics and the extent of binding of Ru species to HSA, Tf and IgG that can be studied by techniques described herein. For the exact identification of mentioned species, other methods should be applied.

### 2.3. Quantification of Separated Ru Species on the CLC Column by the Post-Column ID-ICP-MS

**Figure 4:**
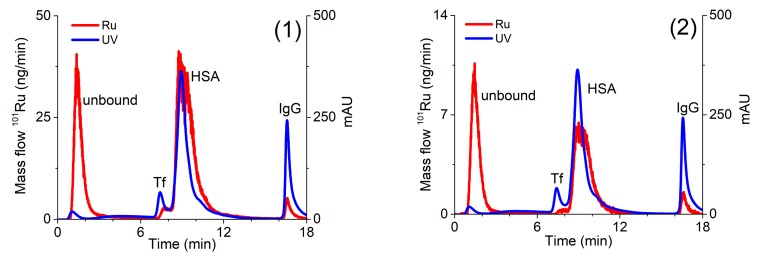
Two-dimensional separation of Ru species in serum sample spiked with complexes (**1**) (4.14 µg/mL Ru) or (**2**) (0.838 µg/mL Ru). Speciation analysis consisted of separation on the CLC monolithic column (24 h after incubation in 5-times diluted serum samples) and on-line UV spectrometry (278 nm) and ICP-MS detection. Ru mass flow is based on measurement of isotope ratios *m/z* 99 and 101.

For the quantification of the Ru species separated on the CLC column, the post-column ID-ICP-MS technique was applied. For this purpose, isotopes at *m/z* 99 and 101 were monitored. To calculate the concentrations of the separated Ru species [41], the mass flow of ^101^Ru was plotted versus time throughout the chromatographic run. Representative chromatograms of serum sample spiked with complexes (**1**) or (**2**) are presented in Figure 4.

### 2.4. Analytical Figures of Merit

#### 2.4.1. Column Recovery

Column recovery was checked by the Ru speciation analysis in five-times diluted human serum spiked with complexes (**1**) or (**2**)**,** after incubation at 37 °C for 24 h, using post-column ID-ICP-MS for quantification of the separated Ru species. Column recoveries were calculated as the ratio between the sum of concentrations of Ru species in the fractions eluted and the Ru concentration in spiked serum injected onto the column. The results are presented in Table 1.

It can be seen that column recoveries were 95% and 104% for serum spiked with complexes (**1**) or (**2**), respectively. These data confirm that the optimized CLC procedure enables quantitative elution of the separated Ru species from the column. 

#### 2.4.2. Repeatability and Reproducibility of Measurement

The repeatability of measurement was evaluated by six consecutive speciation analyses of human serum spiked with complexes (**1**) or (**2**) after incubation for 24 h. Five-time diluted samples were injected onto the CLC columns. The reproducibility of measurement was calculated from a set of six consecutive speciation analyses of the same sample analyzed another day. The data are presented in Table 2.

As can be seen from data of Table 2 good repeatability and reproducibility of measurement were obtained for Ru species separated on the CLC column, with RSDs for particular Ru species ranging from 0.6 to 3.8% and from 1.4 to 6.9%, respectively. 

#### 2.4.3. Limit of Detection, Limit of Quantification and Linearity of Measurement

The limit of detection (LOD) and limit of quantification (LOQ) for the determination of Ru species were calculated as the concentration that provides a signal (peak area) equal to 3 *s* and 10 *s* of a blank sample in the chromatogram, respectively. For the calculation of the LODs and LOQs, eight blank samples of unspiked human serum were injected onto the CLC column. The LODs and LOQs for the separated Ru species are presented in Table 3.

As can be seen, low LODs (<1.6 ng/mL Ru) and LOQs (<5.3 ng/mL Ru) were obtained for the separated Ru species. 

The linearity of measurement, for all the Ru species separated on the CLC column was confirmed over the concentration range from LOQs to 100 ng/mL Ru (correlation coefficient (R^2^) better than 0.997).

The performances of the optimized speciation procedure allow quantitative kinetic studies of the binding of Ru complexes (**1**) and (**2**) to human serum proteins.

### 2.5. Kinetics of the Interaction of Ru Complex *(**1**)* and *(**2**)* With Human Serum Proteins

**Figure 5:**
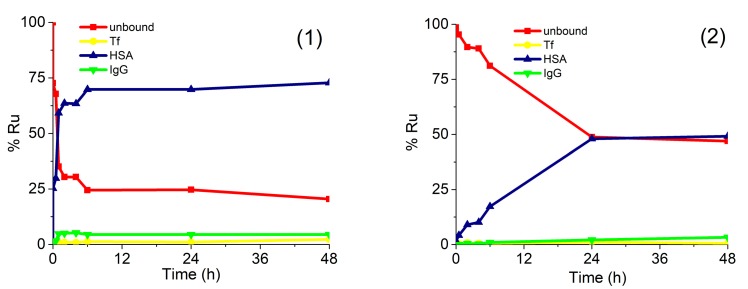
Kinetics of binding of complexes (**1**) and (**2**) to serum proteins. Human serum was spiked with complex (**1**) (0.760 µg/mL Ru) or (**2**) (0.396 µg/mL Ru). For separation of Ru species in 5-times diluted serum samples CLC monolithic column was used. Their separation was followed by the UV spectrometry at 278 nm and post-column ID-ICP-MS detection.

For a better understanding of the drug–protein interaction, kinetic studies of the association of complexes (**1**) and (**2**) with serum proteins were carried out. For this purpose, human serum was spiked with complexes (**1**) or (**2**) in concentration 0.760 µg/mL Ru or 0.396 µg/mL Ru, respectively. Expressed as molar concentrations, the spiking concentration of complex (**1**) was 2.99 mM, while of complex (**2**) 2.60 mM. Similar molar concentrations of complexes (**1**) and (**2**) enabled the comparison of the data from kinetic study. Samples were incubated at 37 °C for 5 min, 30 min, 1 h, 2 h, 4 h, 6 h, 24 h, and 48 h and a speciation analysis was applied under the optimized analytical procedure (Section 3.5). Separated Ru species were quantified by the post-column ID-ICP-MS. The results are presented in Figure 5. 

The data in Figure 5 indicate that both Ru complexes bind mainly to HSA, while their rate of interaction and kinetics are different. Chlorido complex (**1**) rapidly binds to serum proteins. The equilibrium concentrations of Ru species found in human serum were reached 6 h after incubation, with around 70% of complex (**1**) bound to HSA and 5% associated with IgG, while 25% of complex (**1**) remained unbound. It can be further seen that the kinetics and the rate of interaction of complex (**2**) with HSA was much slower and less extensive in comparison to complex (**1**). The equilibrium concentration was obtained 24 h after incubation, with about 50% of complex (**2**) bound to HSA and another 50% as an unbound drug. These data can be compared with the results of protein binding study described by Kladnik et al. [21], where it was investigated the binding of analogous methyl-substituted pyrithione chlorido and pta Ru(II) complexes to BSA. The obtained data reported in this study for chlorido complex (**1**) are in very good agreement with data obtained for its methyl-substituted pyrithione analogue on BSA, as in both cases complexes are bound to albumins in around 70%. Further, complex (**2**) binds to HSA with around 50%, whereas its methyl analogue binds to BSA with around 58%. Taken into consideration small structural changes of both pta Ru complexes and both albumins data from these two studies are comparable as well [21]. New findings have again confirmed more in detail that investigated complexes are partly bound to proteins, but they also exist in their free form. Considering drug design both forms of complexes must be present in the body for an effective biological response. In many cases, proteins, especially HSA, were proven to act as drug carriers in the body to deliver pharmaceutically active agents to the site of actions; however, the unbound form must also be present to trigger pharmacological effect [42]. In this study, we have shown that chlorido and pta complexes possess different rates and extent of the binding to the studied proteins and that various substituents, importantly influence kinetic interactions. Similarly, we have previously observed big differences in the biological activity of another chlorido-pta pair of complexes, namely [(η^6^-*p*-cymene)Ru(4,4,4-trifluoro-1-(4-chlorophenyl)-1,3-butanedione)Cl] and [(η^6^-*p*-cymene)Ru(4,4,4-trifluoro-1-(4-chlorophenyl)-1,3-butanedione)pta]PF_6_. It seems like it is a general trend that chlorido ligand is easier exchanged with water than the pta ligand, i.e., chlorido ligand is more labile than pta. This also affects the kinetics of interactions with biomolecules and thus, also their biological activity [43,44,45]. 

## 3. Materials and Methods

### 3.1. Materials

Ultrapure water (18.2 MΩ cm) was obtained from a Direct-Q 5 Ultrapure water system (Millipore Watertown, MA, USA). All chemicals were of analytical reagent grade. Human serum apo-transferrin (Tf), human serum albumin (HSA) and γ-globulins (IgG) were purchased from Sigma-Aldrich (Steinheim, Germany). 

Merck (Darmstadt, Germany) stock Ru solution (1000 ± 8 mg/L in 7% HCl) was used for the preparation of calibration standard solutions for the determination of the total Ru concentrations in serum samples spiked with Ru-based chemotherapeutics. To control the stability of the ICP-MS, Rh (1000 ± 2 mg/L in 5 % HNO_3_), purchased from Merck, was aspirated along with the sample into the plasma by a peristaltic pump via a T-piece.

For the preparation of buffers and eluents in chromatographic separation 2-amino-2-(hydroxymethyl)propane-1,3-diol (Tris) buffer, sodium hydrogen carbonate (NaHCO_3_), ammonium chloride (NH_4_Cl) and acetic acid (AcOH), purchased from Merck, were used. 

Buffer A consisted of 50 mM Tris-HCl, 30 mM NaHCO_3_ and 10 mM NH_4_Cl, pH 7.4. Buffer B was composed of buffer A + 2 M NH_4_Cl, pH 7.4. Eluent C was 0.5 M AcOH, pH 2. Buffer D was 0.2 M Tris-HCl, pH 7.4. 

Chromatographic supports of CIM Protein G disk was cleaned by AcOH, while of CIM DEAE disk with Merck sodium hydroxide (NaOH) and Merck sodium chloride (NaCl) [35]. 

Complex [(η^6^-*p*-cymene)Ru(1-hydroxypyridine-2(1*H*)-thionato)Cl (**1**) and complex [(η^6^-*p*-cymene)Ru(1-hydroxypyridine-2(1*H*)-thionato)pta]PF_6_ (**2**) were prepared according to the procedure described by Kladnik et al. [21].

Ru-enriched isotope (^99^Ru metallic plate, 15 mg) was obtained from Oak Ridge National Laboratory (Oak Ridge, TN, USA). The declared composition of enriched ^99^Ru plate was 0.12 ± 0.02%, 0.12 ± 0.02%, 97.69 ± 0.1%, 0.74 ± 0.03%, 0.48 ± 0.02%, 0.58 ± 0.01% and 0.27 ± 0.02% for isotopes 96, 98, 99, 100, 101, 102 and 104, respectively. For the preparation of the enriched ^99^Ru stock solution, 15 mg of ^99^Ru metallic plate was dissolved in 1 mL of *aqua regia* and diluted to 10 mL with an appropriate amount of HCl, so that the final concentration of HCl was 7%. The concentration in ^99^Ru stock isotopic spike solution was determined by the reverse ID-ICP-MS procedure [41]. It was found to be 597 ± 10 µg/g ^99^Ru.

### 3.2. Instrumentation

Chromatographic separations were performed on an Agilent (Tokyo, Japan) series 1260 infinity quaternary HPLC pump equipped with a Rheodyne model 7725i (Cotati, CA, USA) injector fitted with 100 µL injection loop and a software controlled six-port valve. An UV-Vis Agilent 1260 infinity series variable wavelength (VWD) detector was used for absorption measurements at 278 nm. For separation of Ru species, one CIM Protein G and one CIM diethylamino (DEAE) monolithic disks, both from BIA Separations d.o.o. (Ajdovščina, Slovenia), were assembled into one housing forming a CLC monolithic column. A CIM disk (dimensions 12 mm i.d and length 3 mm, bed volume 0.34 mL) consisted of a poly(glycidyl methacrylate-co-ethylene dimethacrylate) highly porous monolithic polymer. The CLC column was connected to the HPLC system so that the mobile phase first rinsed the CIM Protein G disk and afterwards the CIM DEAE disk. The outlet of the column was coupled online with an UV-Vis and an Agilent 7900 ICP-MS instrument. ICP-MS was used also for the determination of total Ru concentrations in serum samples. The data were processed with Agilent MassHunter software. The ICP-MS operating parameters were optimized for plasma robustness and for introducing minimum amounts of salts used in the separation procedure (Table S1 in Supplementary Material).

NMR spectroscopy was carried out at room temperature on a Bruker US Avance III 500 spectrometer. ^1^H NMR spectra were recorded at 500 MHz. Chemical shifts are referenced to residual peak of the deuterated solvent D_2_O at 4.79 ppm. Chemical shifts in ^31^P NMR spectra, recorded at 202 MHz, are referenced relatively to an external standard. All NMR data were processed using MestReNova version 11.0.4.

A WTW (Weilheim, Germany) 330 pH metre was used to determine pH. 

A Mettler AE 163 (Zürich, Switzerland) analytical balance was used for weighing. 

### 3.3. Sample Preparation

In order to check the separation of serum proteins on the CLC column, a mixture of standard serum proteins (25 g/L of HSA, 5 g/L of IgG and 2.5 g/L of Tf) was dissolved in buffer A, and diluted 5-times with buffer A before analysis. 

Before the speciation analysis, approximately 5 mg of complex (**1**) was dissolved in 1 mL of MeOH, and appropriately diluted with buffer A, so that the final concentration of MeOH was 0.3%. For the preparation of the complex (**2**) solution, about 5 mg of complex (**2**) was dissolved in 1 mL of water, appropriately diluted with buffer A. 

For NMR stability first D_2_O solution containing 154 mM NaCl was prepared (corresponding to 0.9% (*w/v*) aqueous NaCl solution, thus physiological saline). To 5.0 mg of complex (**1**) 3 µL of MeOD-d_4_ was added and then diluted with 997 µL of D_2_O solution containing 154 mM NaCl. For pta complex (**2**) 1000 µL of D_2_O solution containing 154 mM NaCl was added to 5.0 mg of the sample. After preparation each suspension was filtered (Sartorius Stedim, Minisart® syringe filter, 16553-K, PES 0.1 µm) and 600 µL of the solution was transferred to NMR tube to track the stability. 

Human serum was obtained from a transplanted renal patient by venous puncture after informed consent was obtained. Approximately 300 mL of whole blood was collected into a Pyrex glass container without additives. Blood aliquots were transferred into 20 mL polyethylene tubes and centrifuged for 10 min at 855 *g*. Serum aliquots were transferred to 2 mL polyethylene tubes and stored at −20 °C. Before the analysis, the samples were equilibrated to room temperature. 

For the optimization of the analytical procedure for speciation of Ru complexes (**1**) and (**2**) and for kinetic studies, serum was spiked with complex (**1**) or complex (**2**), so that the final concentration of Ru in spiked samples ranged from 0.396 to 4.14 µg/mL. The samples were incubated at 37 °C from 5 min up to 48 h and diluted 5-times with buffer A before speciation analysis. Five-time sample dilution was necessary due to high serum viscosity and was taking into account in all calculations of Ru concentrations.

### 3.4. Determination of Total Ru Concentration in Spiked Human Serum

Total Ru concentration in spiked human serum was determined in 10-times diluted samples by ICP-MS using standard addition method for calibration.

### 3.5. Chromatographic Procedure

Chromatographic separation was carried out at a flow rate of 1 mL/min. In the first 5 min, isocratic elution with 100% buffer A was applied to elute unbound Ru-based chemotherapeutic. In the next 9 min, linear gradient elution from 100% buffer A to 50% buffer B followed in order to separate Tf from HSA. Then, isocratic elution with 100% eluent C was applied for 4 min to elute IgG. The elution of proteins was followed on-line by the UV spectrometry (278 nm) while the separated Ru species were detected by ICP-MS. To obtain reproducible chromatographic separation it was crucial to effectively regenerate and equilibrate the CLC column. After separation, the column was regenerated with buffer D for 3 min and in the next 3 min with buffer B at a flow rate of 6 mL/min. Finally, the column was equilibrated with buffer A for 5 min at a flow rate 6 mL/min, followed by elution with buffer A for 0.5 min at flow rate of 1 mL/min. The eluents from regeneration and equilibration steps (flow rate 6 mL/min) were directed to waste through a software-controlled six-port valve. After approximately 30 serum separations, the CLC column was dismantled and protein G disk and DEAE disk were cleaned separately [35].

### 3.6. Quantification of Separated Ru Species by Post-Column Isotope Dilution

The separated Ru species were quantified by the post-column ID-ICP-MS technique. Isotopically enriched ^99^Ru was aspirated continuously by a peristaltic pump via a T-piece after the chromatographic separation of Ru species. For the calculation of the concentration of the eluted Ru-species, the mass flow of Ru was plotted versus time during the chromatographic run. Calculations were done by using equations derived for species unspecific post-column ID-ICP-MS analysis [41].

If not stated otherwise, all the experiments were performed in duplicate.

## 4. Conclusions

The CLC monolithic column assembled from one affinity CIM Protein G and one weak anion-exchange CIM DEAE disks was used for separation of Ru complexes (**1**) and (**2**) in human serum. Separated Ru species were monitored on-line by UV spectrometry at 278 nm and ICP-MS at *m/z* 99 and 101. Accurate quantification of separated Ru species was performed by the post-column ID-ICP-MS. The method is selective, robust, repeatable, reproducible (RSD for separated chlorido and Ru species <4 and 7%, respectively), of adequate sensitivity (LODs and LOQs for separated Ru species <1.6 and 5.3 ng/mL Ru, respectively), and demonstrated its potential for studies of the kinetics of bindings of Ru complexes (**1**) and (**2**) to human serum proteins. The data revealed that both Ru complexes interacted mainly with HSA. Complex (**1**) more rapidly and in greater extent binds to HSA than complex (**2**). The equilibrium concentration, in which around 70% of complex (**1**) is bound to HSA, 5% is associated with IgG and the rest 25% remains as a free drug, was reached 6 h after incubation. Interaction of complex (**2**) with HSA was much slower and less extensive than with complex (**1**). The equilibrium concentration was obtained 24 h after incubation, with about 50% of complex (**2**) bound to HSA, while 50% of complex (**2**) remained unbound. This detailed study on binding kinetics of (**1**) and (**2**) suggests that bound and unbound forms of complexes are present in human serum, which is an important finding considering drug design, where pharmacokinetics as well as pharmacodynamics properties must be well defined. 

## Figures and Tables

**Table 1:** Concentrations of Ru species in human serum spiked with complexes (**1**) or (**2**). Ru species were separated on CLC column and their concentration determined by ICP-MS, using post-column ID-ICP-MS. Column recovery was calculated as the ratio between the sum of concentrations of Ru species in the fractions eluted and the Ru concentration in spiked serum injected onto the column.

Complex	Ru Injected (µg/mL)	Unbound Ru (µg/mL)	Ru-Tf (µg/mL)	Ru-HSA (µg/mL)	Ru-IgG (µg/mL)	Ru Eluted (µg/mL)	Column Recovery (%)
**(1)**	4.14 ± 0.08	1.28 ± 0.01	0.038 ± 0.001	2.48 ± 0.02	0.125 ± 0.002	3.92 ± 0.001	95 ± 1
**(2)**	0.838 ± 0.025	0.386 ± 0.004	0.014 ± 0.001	0.443 ± 0.005	0.027 ± 0.001	0.869 ± 0.008	104 ± 3

**Table 2:** Repeatability and reproducibility of measurement for speciation of Ru in serum sample spiked with complexes (**1**) (4.14 µg/mL Ru) or (**2**) (0.838 µg/mL Ru) on CLC column.

	Complex (1)		Complex (2)	
Ru species	Repeatability RSD (%)	Reproducibility RSD (%)	Repeatability RSD (%)	Reproducibility RSD (%)
Unbound Ru	1.6	6.9	1.2	4.4
Ru-Tf	3.3	5.2	1.6	6.3
Ru-HSA	0.63	1.4	2.9	3.3
Ru-IgG	2.7	4.9	3.8	4.0

**Table 3:** Limits of detection (LOD) and limits of quantification (LOQ) for separated Ru species on the CLC monolithic column with ICP-MS detection.

Ru Species	LOD (ng/mL Ru)	LOQ (ng/mL Ru)
Unbound	0.32	1.1
Ru-Tf	0.12	0.40
Ru-HSA	1.6	5.3
Ru-IgG	1.1	3.6

## Supplementary Materials

Table 1S. ICP-MS operating parameters. Figures S1–S3. NMR stability spectra.
