# Supplementary material for: Binding Kinetics of Ruthenium Pyrithione Chemotherapeutic Candidates to Human Serum Proteins Studied by HPLC-ICP-MS
Source: Molecules. 2020 Mar 26;25(7):1512. doi: 10.3390/molecules25071512 (PMC7180866; doi:10.3390/molecules25071512)
Supplement: Supplementary file 1 [file molecules-25-01512-s001.pdf]

Supplementary Material

Binding Kinetics of Ruthenium Pyrithione Chemotherapeutic Candidates to Human Serum Proteins Studied by HPLC-ICP-MS

Katarina Marković [1,2], Radmila Milačič [1,2], Stefan Marković [1,2], Jerneja Kladnik [3], Iztok Turel [3,*] and Janez Ščančar [1,2,*]

[1] Department of Environmental Sciences, Jožef Stefan Institute, Jamova 39, SI-1000 Ljubljana, Slovenia; katarina.markovic@ijs.si (K.M.); radmila.milacic@ijs.si (R.M.); stefan.markovic@ijs.si (S.M.)

[2] Jožef Stefan International Postgraduate School, Jamova 39, SI-1000 Ljubljana, Slovenia

[3] Faculty of Chemistry and Chemical Technology, University of Ljubljana, Večna pot 113, SI-1000 Ljubljana, Slovenia; Jerneja.Kladnik@fkkt.uni-lj.si (J.K.); Iztok.Turel@fkkt.uni-lj.si (I.T.)

* Correspondence: janez.scancar@ijs.si (J.Š.) Tel.: +386-1-477-3846; Iztok.Turel@fkkt.uni-lj.si (I.T.) Tel.: +386-1-479-8525

Table 1S. ICP-MS operating parameters.

Parameter	Type/Value<br>Ru speciation analysis	Type/Value<br>Analysis of total Ru content
Sample introduction		
Nebuliser	Miramist	Miramist
Spray chamber	Scott	Scott
Skimmer and sampler	Ni	Ni
Plasma conditions		
Forward power	1550 W	1550 W
Plasma gas flow (Ar)	15.0 L min^{-1}	15.0 L min^{-1}
Carrier gas flow (Ar)	0.45 L min^{-1}	1.05 L min^{-1}
Dilution gas flow (Ar)	0.75 L min^{-1}	/
Make up gas flow (Ar)	/	0.10 L min^{-1}
Total carrier gas flow	1.20 L min^{-1}	1.15 L min^{-1}
He gas flow	4.3 mL min^{-1}	4.5 mL min^{-1}
Omega bias	-110 V	-120 V
Cell entrance	-40 V	-40 V
Cell exit	-60 V	-60 V
Deflect	3.8 V	3.0 V
Plate bias	-60 V	-60 V
Sample uptake rate	1.0 mL min^{-1}	0.3 mL min^{-1}
Data acquisition parameters		
m/z of isotopes monitored	^{99}Ru, ^{101}Ru	^{101}Ru
m/z of internal standards*	^{103}Rh	^{103}Rh
Total acquisition time	1080 s	/

*In speciation analysis, internal standards were not used when the ID-ICP-MS procedure was applied

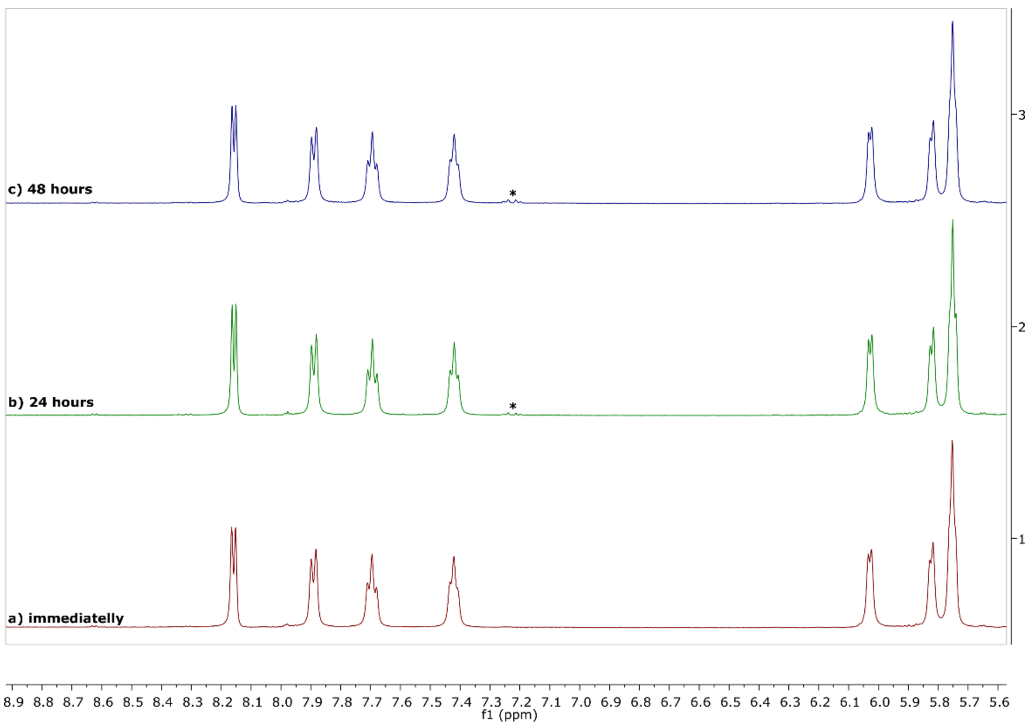

Figure 1. ^{1}H-NMR stability spectra of the chlorido complex (**1**) in 0.3% MeOD-d_4/D_2O solution containing 154 mM NaCl recorded a) immediately, b) after 24 hours and c) after 48 hours. The release of *p*-cymene is labelled with an asterisk (*).

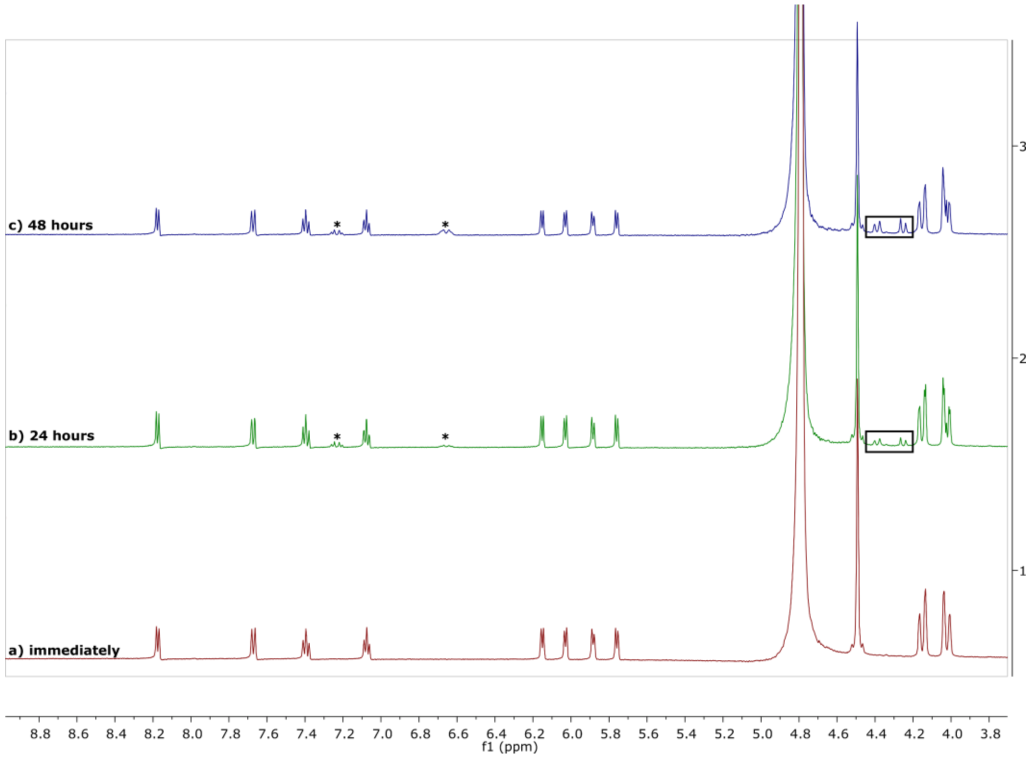

Figure S2: ^{1}H-NMR stability spectra of the pta complex (**2**) in D_2O solution containing 154 mM NaCl recorded a) immediately, b) after 24 hours and c) after 48 hours. The release of *p*-cymene is labelled with an asterisk (*) and ptao with squares.

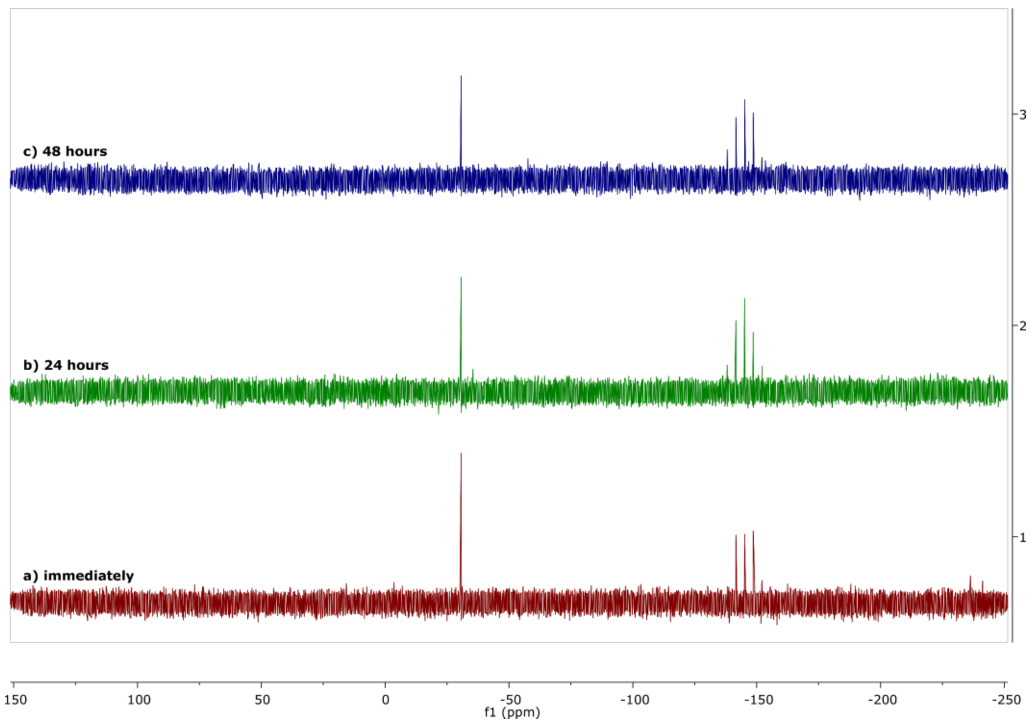

Figure S3: ^{31}P-NMR stability spectra of the pta complex (**2**) in D_2O solution containing 154 mM NaCl recorded a) immediately, b) after 24 hours and c) after 48 hours.
